# Transformation of fibroblast‐like synoviocytes in rheumatoid arthritis; from a friend to foe

**DOI:** 10.1186/s13317-020-00145-x

**Published:** 2021-02-05

**Authors:** Mohammad Javad Mousavi, Jafar Karami, Saeed Aslani, Mohammad Naghi Tahmasebi, Arash Sharafat Vaziri, Ahmadreza Jamshidi, Elham Farhadi, Mahdi Mahmoudi

**Affiliations:** 1 Rheumatology Research Center, Tehran University of Medical Sciences, Tehran, Iran; 2 Department of Immunology, School of Medicine, Tehran University of Medical Sciences, Tehran, Iran; 3 Department of Hematology, Faculty of Allied Medicine, Bushehr University of Medical Sciences, Bushehr, Iran; 4 Department of Immunology, School of Medicine, Iran University of Medical Sciences, Tehran, Iran; 5 Department of Laboratory Sciences, Khomein University of Medical Sciences, Khomein, Iran; 6 Joint Reconstruction Reseach Center, Tehran University of Medical Sciences, Tehran, Iran; 7 Inflammation Research Center, Tehran University of Medical Sciences, Tehran, Iran

**Keywords:** Cancer‐like behavior, Rheumatoid arthritis, Fibroblast‐like synoviocyte, Genetics, Epigenetics

## Abstract

Swelling and the progressive destruction of articular cartilage are major characteristics of rheumatoid arthritis (RA), a systemic autoimmune disease that directly affects the synovial joints and often causes severe disability in the affected positions. Recent studies have shown that type B synoviocytes, which are also called fibroblast-like synoviocytes (FLSs), as the most commonly and chiefly resident cells, play a crucial role in early-onset and disease progression by producing various mediators. During the pathogenesis of RA, the FLSs’ phenotype is altered, and represent invasive behavior similar to that observed in tumor conditions. Modified and stressful microenvironment by FLSs leads to the recruitment of other immune cells and, eventually, pannus formation. The origins of this cancerous phenotype stem fundamentally from the significant metabolic changes in glucose, lipids, and oxygen metabolism pathways. Moreover, the genetic abnormalities and epigenetic alterations have recently been implicated in cancer-like behaviors of RA FLSs. In this review, we will focus on the mechanisms underlying the transformation of FLSs to a cancer-like phenotype during RA. A comprehensive understanding of these mechanisms may lead to devising more effective and targeted treatment strategies.

## Background

Rheumatoid arthritis (RA) is a complex, systemic, and chronic autoimmune disorder that is recognized as the most common inflammatory arthropathy worldwide [1]. Although the disease can occur in all individuals and all age ranges, the prevalence is highest among women over 40 years. The hallmark of the disease is a chronic inflammation of the joints that is characterized by synovial membrane hypertrophy, hyperplasia, and symmetrical polyarthritis. The inflammation may destroy cartilage, bone, and formation of extra-articular manifestations (EAMs) [2], which ultimately leads to limitations, disabilities, loss of function [2, 3], loss of quality of life [4], and increased morbidity and mortality [5]. Similar to most autoimmune diseases, RA is also a multifactorial disease. Based on studies on genetic predisposition in twins and families, the overall risk associated with genetic factors and heritability of RA is about 50–60% [6, 7]. Besides, cigarette smoking is the most important environmental factor involved in the disease onset, especially in people with a predisposing genetic background [8].

**Fig. 1:**
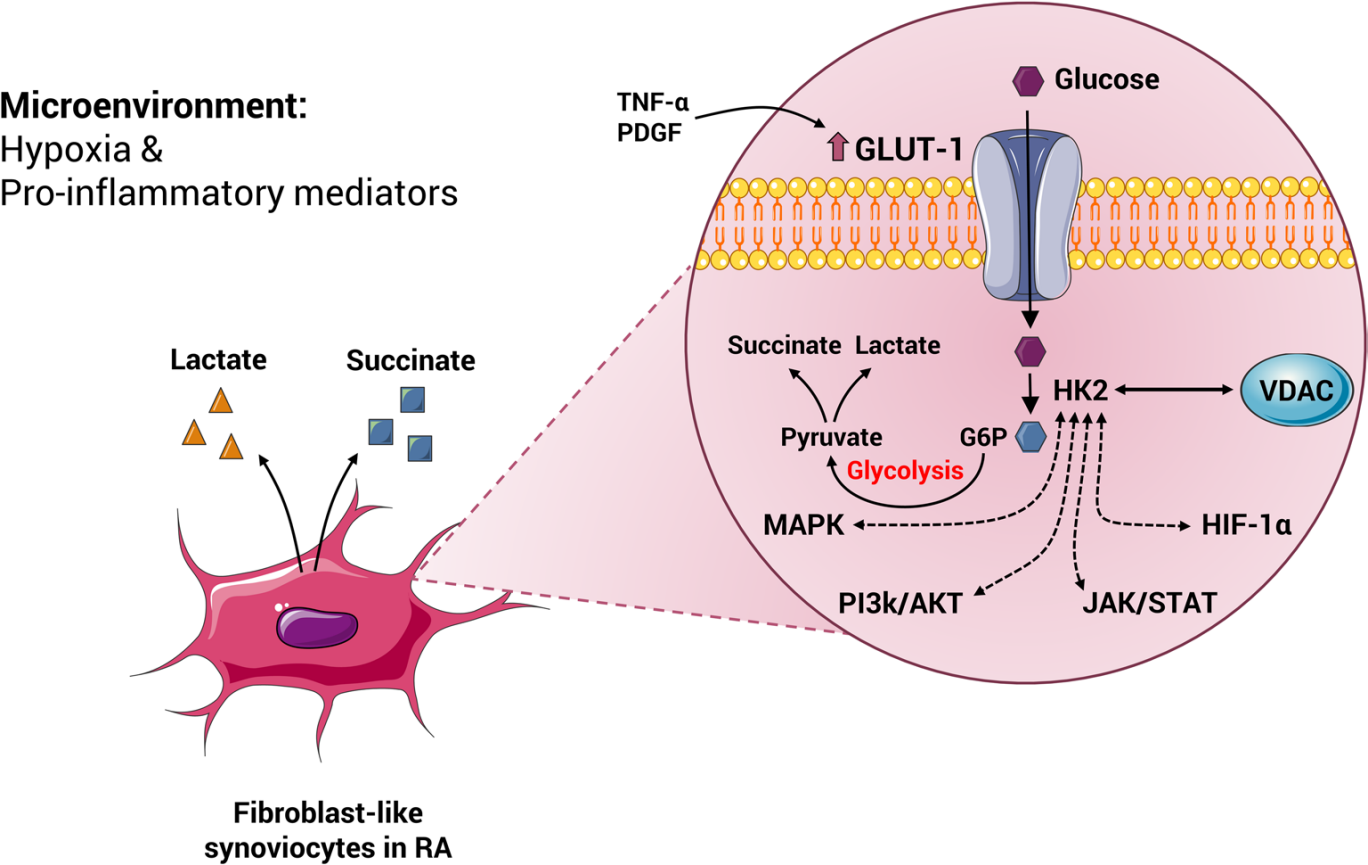
Three main axes of tumor-like behaviors of RA FLSs; metabolism, genetics and epigenetics modifications

Specific fibroblasts located in the synovial membrane of the joints are referred to as synovial fibroblasts (SFs) or fibroblast-like synoviocytes (FLSs) [9, 10], which is also referred as B-type synoviocytes versus A-type synoviocytes (macrophage-like synoviocytes; MLSs). In recent years, FLSs have been introduced as the key players in the RA synovium [9, 11]. FLSs are considered as the main source of cartilage degradation factors and inflammation [11]. In a normal physiological state, FLSs play an essential role in the homeostasis of the joints. Moreover, they play a chief role in releasing necessary synovial fluid components. Conversely, in activated state and joint inflammatory conditions, FLSs lose their contact inhibition potential, alongside with high proliferation, reduced apoptosis, and increased expression of cytokines and chemokines, adhesions molecules, and matrix metalloproteinases (MMPs), which contribute to pannus formation as a critical player [10, 12]. Synovial hyperplasia is frequently seen in RA synovium, which results from the accumulation of FLSs and MLSs in a specific inflammatory microenvironment [12–14]. RA FLSs display cancerous properties in inflamed synovial tissue, whereby they exhibit local tumor-like destructive and invasive characteristics [15, 16]. The phenotype, behavior, and role of FLSs in RA resemble the fundamental role of cancer-associated fibroblasts (CAFs) in various cancers [17, 18]. In this article, we aimed to review the FLS transformation in RA, tumor-like behaviors of RA FLSs, and discuss the underlying mechanisms in three principal axes of metabolism, genetics, and epigenetic modifications (Fig. 1).

## FLSs and RA pathogenesis

FLSs are mesenchymal origin cells and particularly found in the synovium lining membrane, that exhibit many features in common with fibroblasts, such as the expression of type IV and V collagens, and vimentin. On the other side, these cells also express specific surface markers, including Thy-1 membrane glycoprotein (CD90), decay-accelerating factor (DAF; CD55) and secrete unique proteins, like lubricin and hyaluronic acid [synthesized by UDP-glucose 6-dehydrogenase (UDPGDH)] [9, 10]. Concerning cell adhesion, FLSs express vascular cell adhesion molecule-1 (VCAM-1) and cadherin-11 [19]. Cadherin-11, one of the major FLS markers, play a key role in activating and regulating the function of FLSs in addition to cell adhesion [20]. The concept that FLSs destroy the cartilage and supporting structures of the joint is supported by data in cadherin-11 knockout mice, in which the animal lacks an intimal lining, regardless of normal development. Despite an inflammatory state as well as the progression of bone destruction, these synoviocyte-deficient mice do not present arthritis-induced cartilage damage [21].

The intimal lining of the RA synovium displays remarkable changes, with a marked increase in cellular content, and the lining membrane of the RA synovium expands 10–20-fold FLSs [22]. MLSs in RA synovium display a highly activated phenotype and produce pro-inflammatory cytokines, chemokines, and growth factors. These products can activate local FLSs in the lining and induce them to produce their mediators, especially interleukin (IL)-6, prostanoids, and MMPs. This process establishes a paracrine/autocrine network that leads to synovitis, recruitment of further immune cells to the joint, and contribution to the destruction of the extracellular matrix. Among these, interactions between FLSs and synovial T lymphocytes have been reported strongly *in vitro* and *in vivo* [23, 24]. T lymphocytes, even in the resting state, have the ability to activate the FLSs and stimulate the production and secretion of inflammatory mediators such as IL-6, IL-8, and prostaglandin E2 (PGE2) from them [25]. On the other hand, FLSs can also play an effective role in activating, presenting super antigens and proliferative stimulation to T lymphocytes [26].

The hypertrophied synovial tissue, called ‘pannus’ at the cartilage-bone interface, masks the cartilage and erodes it towards the bone. The pannus, which is composed of macrophages, osteoclasts, and invasive FLSs with relatively few lymphocytes, behaves like a locally invasive tumor [27]. With regards to FLSs’ aggressive properties, hyperplasia, and production of vast amounts of proteases, these cells act as primary players in bone and cartilage destruction in the inflamed joints [22].

## The transformed phenotype of RA FLSs

During RA development, the phenotype of RA FLSs is altered to an invasive and aggressive form, with increased migration ability and reduced attachment-dependent growth, leading to intimal lining layer hyperplasia and the articular cartilage destruction and, ultimately, pannus formation [9, 10]. Transformed FLSs in the inflammatory microenvironment of the RA synovium are not just “passive responders”, but as “imprinted aggressors” can continue to change their behaviors and phenotype, independently of external stimulation of the existing toxic and inflamed milieu [10, 28]. It appears that FLSs are inherently and permanently transformed, similar to the transformation of cancer cells. In this regard, it has been shown that this transformed phenotype of RA FLSs persists upon transplantation to severe combined immunodeficiency (SCID) mouse model and this engraftment culminated in articular cartilage modulation through extracellular matrix destruction. Nonetheless, this behavior has not been observed in FLSs isolated from healthy subjects or even cases with osteoarthritis (OA) [29]. Significant differences in FLSs differentiation, proliferation, and adhesion are involved in divergences in the patterns and signature of DNA methylation in FLSs isolated from early RA individuals compared to that of patients with established RA [30]. Remarkably, studies that illustrate the role of FLSs in synovitis transition from an affected to a non-affected joint remind a process known as “metastasis”, an important characteristic of cancerous cells pathogenesis [29, 31, 32].

### Cell metabolism in the transformed RA FLSs

One of the main metabolic hallmarks in RA patients is a significant increase in “calorie consumption” which resulted in increased heat in the involved joint. Following extensive studies to detect metabolic and mitochondrial changes in tumor cells of various malignancies, studies on the metabolic changes of immune cells, stromal cells, and fibroblasts in dysregulated and inflammatory conditions have been performed [33–37]. Daily intake of energy increases in RA as it has been reported an 8% higher basal metabolic rate (BMR) in non-smoker RA patients compared to healthy subjects (this increase was even 20% in smoker RA subjects) [38]. Increased disability, decreased quality of life, and early mortality of RA patients have also been attributed to the increased metabolism rate [39].

In the transformed FLSs, metabolism of the four main classes of macromolecules (carbohydrates, lipids, proteins, and nucleic acids) changes, conferring an invasive potential to these cells [40, 41]. In general, glucose metabolism (i.e., glycolysis and pentose phosphate pathway), tricarboxylic acid (TCA) cycle, and amino acid metabolisms, such as tyrosine-derived catecholamine and protein biosynthesis, in RA FLSs are severely impaired even compared to OA FLSs [42–44]. Investigation of the metabolic profile of RA synovial tissue has shown the main shift toward glucose and choline metabolism in RA FLSs [45–47].

**Fig. 2:**
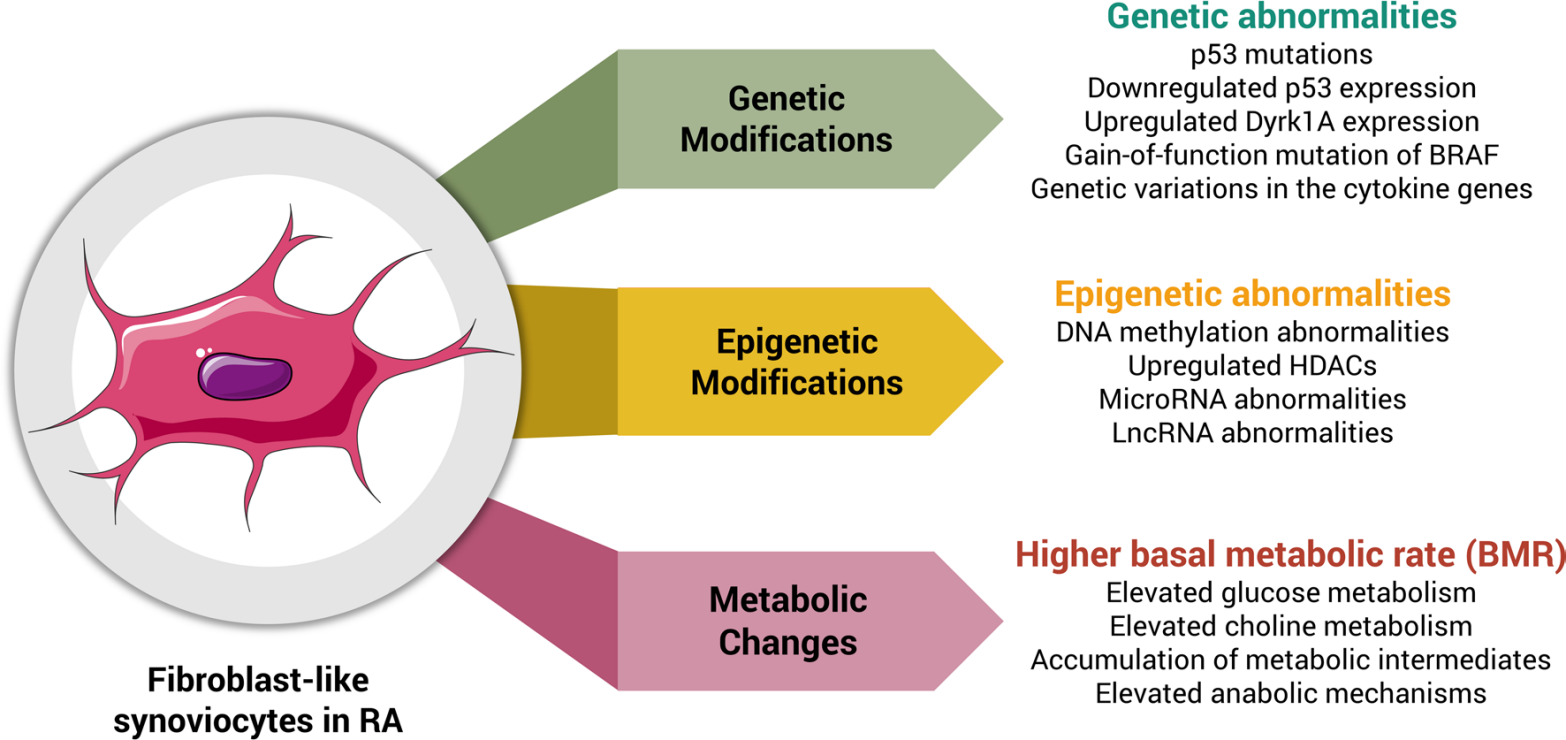
Increased and chronic glucose metabolism in RA-FLSs due to the presence in hypoxic microenvironment and abundant inflammatory mediators in the inflamed synovium

Elevated glucose metabolism is one of the main characteristics of the active and proliferating cells, similar to tumor cells. Increased metabolism of glucose and glycolysis process provides the required amounts of metabolic mediators to support the anabolic processes of lipids, nucleic acids, and proteins in proliferating RA FLSs [48]. Upregulated glucose metabolism in RA FLSs can contribute to inflammation and joint damage. In addition to overexpression of glucose transporter 1 (GLUT1) and glucose uptake in RA FLSs, tumor necrosis factor-α (TNF-α) and platelet-derived growth factor (PDGF) induce the GLUT1 mRNA expression and glucose metabolism. On the other hand, glucose deprivation or inhibition of glycolysis by glycolytic inhibitors such as bromopyruvate (BrPa), disrupts the proliferation, migration, and cytokine secretion of RA FLSs. Additionally, *in vivo* experiments have established that inhibition of glycolysis strongly reduced the severity of arthritis [44]. Elevated chronic glucose metabolism due to hypoxia and highly pro-inflammatory mediators in the microenvironment of the RA synovium, stimulates and activates important signaling pathways in FLSs, including mitogen-activated protein kinase (MAPK), phosphatidylinositol-3-kinase (PI3K)/Akt, Janus kinase (JAK)/STAT, and hypoxia-inducible factor (HIF)-1α [49]. Eventually, upregulated glucose metabolism in inflammatory conditions and arthritis-like RA resulted in increased serum lactate and succinate [50, 51] (Fig. 2).

Raised levels of choline kinase (CK)-α (also known as choline phosphokinase), the primary enzyme of the choline pathway and phosphatidylcholine synthesis, has been shown to play a fundamental role in cell transformation, invasion, and metastasis in various cancers [52–54]. CK is highly expressed in the RA synovial tissue and cultured RA FLSs. So, CK inhibitors inhibit invasive properties, including cell migration and resistance to apoptosis of FLSs *in vivo*, as well as a deceleration of arthritis development and amelioration of arthritis symptoms [47].

It has been documented that hypoxia can promote a switch to glycolysis, supporting abnormal angiogenesis, cellular invasion, and pannus formation [55]. In addition, glycolytic metabolites, including succinate [56], α-ketoglutarate, fumarate, and acetyl-CoA, are involved in changes in epigenetics and cell signaling [57]. Furthermore, the accumulation of metabolic intermediates in FLSs under stress and hypoxic conditions contributes to anabolic mechanisms of nucleic acids, lipids, and proteins [41]. Interestingly, it has been reported that as RA progresses due to impairment in the lipids and glucose metabolism, the patients might be susceptible to the co-occurrence of cardiovascular diseases (CVD) and metabolic syndrome [58].

### Hyperplasia and resistance to apoptosis in RA FLSs

Increased proliferation of RA FLSs is one of the causes of RA synovial hyperplasia [59]. However, expression of proto-oncogenes, such as c-myc in FLSs, lack of mitosis as well as immunohistochemistry (IHC) staining for some markers of nuclear division, suggests that cell division may not be the leading cause of synovial hyperplasia [41]. The limited number of mitotic divisions, the expression of Ki-67 as cell cycle marker, or the expression of proliferating cell nuclear antigen (PCNA), all suggest that DNA synthesis may predominantly not occur in RA FLSs [22].

In addition to proliferation, apoptosis is another mechanism to control cell numbers in response to DNA damage. It has been shown that significant changes occur in the mitochondrial pathways of apoptosis in RA FLSs, which leads to outstanding resistance to apoptosis. These changes include dysregulation of the Bcl-2 family proteins, which plays a major role in the regulation of the intrinsic pathway of apoptosis [60, 61] as well as p53 mutations, that explains the absence of apoptosis due to p53 impairments [62, 63]. It has been shown that inactivation of the p53 protein by papillomavirus E6 protein in FLSs leads to increased growth rate and inhibited the apoptosis process [64].

Along with the established mechanisms involved in the regulation of apoptosis, it has been determined that the essential glycolytic enzymes can translocate to the nucleus, where they can play an anti-apoptotic role independently of their canonical metabolic role. Hexokinase 2 (HK2), which plays a typical role in the production of glucose 6-phosphate from glucose, can attach to the mitochondrial outer membrane through a transaction with the voltage-dependent anion channel (VDAC) protein [65]. The interaction between HK2 and VDAC prevents the release of pro-apoptotic molecules and inhibits cell apoptosis. Interestingly, there is an overexpression of HK2 in RA FLSs compared to OA FLSs [44].

### Dysregulation of angiogenesis in RA FLSs

Hypoxia is the primary mechanism for promoting the formation and development of new vessels [66, 67]. RA synovial hyperplasia, due to increased oxygen consumption, causes an intensive hypoxic environment. Although the oxygen tension is up to 8% in healthy conditions, it is less than 1% in the toxic and highly hypoxic milieu of RA synovium [68]. Hypoxia results in HIF-α activation and overexpression, which induces the transcription of HIF-responsive genes. Among these genes, vascular endothelial growth factor (VEGF), fundamentally participates in synovial angiogenic processes, development of synovitis, and the RA pathogenesis [69].

Other angiogenic growth factors like fibroblast growth factor (FGF) and angiopoietin-2 are also secreted mainly by activated synovial FLSs and MLSs. Furthermore, hypoxia activates glycolytic enzymes like glucose 6-phosphate isomerase (G6PI) as well as glycolysis [70], which subsequently leads to the increased production and secretion of glycolytic intermediate components (like succinate and lactate). These components are potent angiogenic stimulants that play a useful role in sustaining the process of angiogenesis [55, 71]. The angiogenesis, which means the formation of new vessels, has been implicated as one of the fundamental mechanisms in the pathogenesis of several autoimmune diseases including RA, OA and spondyloarthropathies. In RA, this process plays a key role in the over-migration and extravasation of the circulating immune cells to the inflammatory and hypertrophic joints and the supply of nutrients and oxygen needed in their toxic environment [72, 73].

### Migration, invasion, and metastasis of RA FLSs

*In vitro* experiments have indicated that RA FLSs play a prominent role in the cartilage tissue destruction, upon being co-cultured with the macrophage line U937. This finding demonstrated that the migration and invasion of FLSs had a fundamental role in the destruction of cartilage and the pannus formation in RA [74]. It has also been further reported that when FLSs isolated from RA patients are transplanted into SCID mice, display aggressive and destructive characteristics in the absence of other immune cells [29]. The initial binding of FLSs to the extracellular matrix components, namely proteoglycans and collagens, results in the activation of signaling pathways involved in their invasive behavior. Subsequently, it has been shown that integrins, particularly the β1 family, are overexpressed in RA FLSs. Blocking these integrins on the surface of RA FLSs decreases the binding strength and their invasive capacity [75–77]. Communication and bilateral regulation of integrin activation and cell metabolism is an emerging paradigm. Various metabolic pathways, such as AMP-activated protein kinase (AMPK), mammalian target of rapamycin complex 1 (mTORC1), and HIF-1α are involved in the regulation of integrins’ transcription, function and expression on the cell surface [78, 79]. On the other hand, integrins bilaterally regulate various metabolic pathways by linking to these pathways or directly interacting with metabolic enzymes [80].

Class 3 semaphorins genes, with their inhibitory role in the progression, migration, and invasion of various cancers, were severely reduced in the synovium of RA patients. In particular, semaphorin-3B and semaphorin-3F reduce the migratory and invasive capacity of RA FLSs and suppress the expression of MMPs, thus being proposed as therapeutic targets in suppressing the invasive behavior of RA FLSs [81].

Platelet-derived growth factor (PDGF) levels in the RA synovium and synovial fluid has been plentifully reported [82–84]. In the pathogenesis of RA, PDGF is considered to be an important growth factor and stimulate of production and secretion of the inflammatory mediators of FLSs, in synergistic association with transforming growth factor-beta (TGF-β) [83, 85]. PDGF is essential for the formation of hypertrophic FLS architecture and the extracellular matrix-degrading invadosome, invasion to cartilage, via activation of several signaling pathways, particularly the PI3K/AKT pathway [86]. Also, the intimal lining has been identified as the primary source of MMPs in RA, and is able to active FLSs with the secretion of collagenases and metalloproteinases, such as MMP-2 and MMP-9, and initiate the destruction of ECM and cartilage [87, 88].

In a recent comprehensive study on the role of different immunological and non-immunological pathways in the behavior of RA FLSs, unexpectedly, the “Huntington’s Disease Signaling” pathway and specifically huntingtin-interacting protein-1 (HIP1) was introduced as the regulator of matrix invasion in RA FLSs [89]. Previous studies have also shown that HIP1 is associated with cell movement and invasion in cancers [90, 91].

## Genetic abnormalities involved in FLS cancer‐like behavior

The most critical genetic abnormalities occurred in the context of cancer-like behavior of RA FLSs are those that are also seen in tumors. For example, the p53 tumor suppressor gene has been observed to harbor mutations that contribute to cancerous consequences, including enhanced invasion and proliferation. Inazuka et al. found that there was a p53 mutation with plausible functional modification in four (44.4%) of the nine RA patients. Moreover, 10 cDNA subclones were identified to harboring ten p53 mutations, in which eight of them were associated with amino acid changes or truncated protein. Functional mutations of p53 were reported in the substitution of Gly at amino acid residue 245 to Asp in two RA patients in three subclones. This mutation was suggested to may functionally affect the FLS, but the evidence was not strongly supportive of the role of p53 mutation in RA [92]. Nonetheless, P53R248Q mutation was reported to downregulate the expression of the pro-apoptotic molecule p53AIP1 in the MH7A RA FLS cell line, resulting in a decreased apoptosis rate and, increasing the tumor-like proliferation of these cells [93]. It seems that p53 mutations are induced by genotoxic exposure randomly, which may be seen in a small number of RA synoviocytes that were present in both erosive and non-erosive areas of the synovium. Moreover, the most important contributing factor for invasive behavior of the cells might be the vicinity of bone and cartilage instead of harboring a p53 mutation [94]. On the other hand, it should be noted that p53 positive cells might not be present in all of the synovial tissues of RA subjects [95]. Nonetheless, several other studies have reported upregulated expression of p53 in RA FLSs [96–98]. In line with these observations, abnormalities were identified in the p53 in RA patients; nonetheless, they were not associated with the cancerous characteristics of the rheumatoid synovium, as evaluated by proliferative manifestations [99].

Except than p53 tumor suppressor gene, other genetic abnormalities have also been reported to be involved in the cancerous behavior of FLSs. Dual-specificity tyrosine-regulated kinase 1A (Dyrk1A), a regulator of the MAPK pathway activation, plays a role in the proliferation and differentiation of mammalian cells. An overexpression of Dyrk1A was observed in the RA synovial tissues as well as in a TNF-α-induced FLSs activation model. It was demonstrated that Dyrk1A might increase the cancerous traits of FLSs, including proliferation, migration, and invasion through suppressing the expression of sprouty RTK signaling antagonist 2 (Spry2) and activating the MAPK/ERK signaling pathway in RA [100]. B-Raf proto-oncogene, serine/threonine kinase (BRAF) is also involved in the regulation of the MAPK/ERK signaling pathway and finally impresses the cell division and differentiation. The V600R gain-of-function mutation of BRAF, which has been associated with several cancers, was also identified in the RA synovial fibroblasts. However, only two of the nine evaluated subjects indicated this mutation. V600R mutation plays a role in the fibroblast transformation, which in turn was involved in the destruction of articular cartilage and bone in RA [101].

Aberrant activation of the sonic hedgehog (SHH) signaling pathway plays a vital role in tumor formation and development. Besides, the SHH signaling pathway through the MAPK/ERK pathway was found to regulate proliferation, migration and tumor-like behavior of RA FLSs [102]. In this regard, it was suggested that targeting the SHH pathway can be considered as an effective therapeutic strategy in RA patients.

Genetic variations in the cytokine genes have also been evaluated with respect to the cancerous behaviors of the RA FLSs. The IL-10 genotype, which is associated with RA progression, did not influence the invasiveness of the RA FLSs when the − 2849 non-G carriers were compared to those who were G carriers. However, the adenoviral gene transfer of IL-10 to FLSs resulted in the inhibition of FLS invasiveness [103].

## Epigenetic abnormalities involved in cancer‐like behavior of FLS

Epigenetics is characterized as stable and heritable alterations in gene expression without changes in the DNA sequence [104]. Epigenetic modifications (DNA methylation, histone modifications, and non-coding RNAs), modulate gene expression patterns during normal physiological processes and also in response to environmental factors (Table 1). The cellular phenotype and function could be modulated through various epigenetic patterns, named the epigenetic signature. There are many epigenetic modifications in the RA FLSs, which are capable of being involved in RA pathogenesis [105].

**Table 1:** Epigenetic abnormalities of the RA FLS concerning cancer-like behavior

Name	Status	Function	References
PTPN11	Hypomethylated	Migration	[113]
LINE1	Hypomethylated	Aggressiveness	[114]
HDAC1 and 2	Upregulated	Apoptosis and proliferation	[117]
miR-10a	Downregulated	Migration, proliferation, invasion	[128, 129]
miR-27a	Downregulated	Invasion and migration	[130, 131]
miR-30a	Downregulated	Autophagy	[132]
miR-34a	Downregulated	Apoptosis	[133, 147]
miR-15a	Downregulated	Apoptosis	[134]
miR-124a	Downregulated	Proliferation	[132]
miR-155	Downregulated	The proliferation and invasive behavior	[136, 139]
miR-126	Upregulated	Apoptosis	[135]
miR-221	Upregulated	Invasion and migration	[140]
miR-663	Upregulated	Proliferation	[141]
miR-21	Upregulated	Proliferation	[142]

### DNA methylation abnormalities

It has been documented that the methylation pattern of the FLS cells is different in RA and OA patients [106]. It is important to notice that DNA methylation and transcription patterns are different in the RA FLS cells isolated from various joints. These findings indicate that there is a complex and joint-specific relationship between gene expression and DNA methylation in modulating joint inflammation at different anatomical locations [107]. Subsequently, investigation on the peripheral blood (PB) lymphocytes in RA patients showed that the genome is hyper-methylated. Therefore, the DNA methylation signature of PB-lymphocytes could be considered as a risk indicator [108]. Genes with different methylation pattern in PB-lymphocytes are involved in focal adhesion, cell migration, and extracellular matrix interactions, which resulted in chronic inflammation in RA [109]. Analysis of methylation patterns showed that the differences are seen in integrin, PDGF, and Wnt pathway genes and propose that cell imprinting linked to adhesion, differentiation, and proliferation changes through the disease development [110].

Limb bud and heart development (LBH) deficiency lead to an increase in DNA damage and cell cycle arrest in the S-phase. Furthermore, in vivo, LBH deficiency causes abnormalities in the cell cycle and increases the severity of arthritis in a mouse model [111]. A combination of the differentially methylated loci (DML) with a risk variant regulates the LBH expression and function, elucidating how epigenetic mechanisms and genetic risk can interact to change cellular function [112]. Tyrosine-Protein phosphatase non-receptor type 11 (*PTPN11*), encodes the protein tyrosine phosphatase SHP2, showed an elevated expression level in the RA FLSs and played a critical role in the migration of the cells. It has been shown that low expression of SHP2 in the serum transfer mouse model is associated with low disease activity, demonstrating that it could be a potential therapeutic target. Methylation of an intronic enhancer of PTPN11 could increase the expression of SHP2 in the RA FLSs [113].

It has been shown that there is an increased expression (30–300 fold) in long interspersed element 1 (LINE1) in the RA FLSs, compared with the normal FLSs, and its expression is regulated by the DNA methylation mechanism [114]. Investigations on the RA FLS cells propose that LINE1 is associated with the aggressive phenotype of these cells through regulation of stress-activated protein kinase (SAPK) [p38 MAPK] signaling cascades, which is a part of a kinase pathway that leads to the production of cytokines including IL-8, IL-6 [115], and MMPs [116].

### Histone modifications

In histone deacetylase (HDACs) evaluation, two HDACs; HDAC1 and − 2, were upregulated in the RA FLSs compared with the OA FLSs. It has been reported that knockdown of these two HDACs might lead to low proliferation and high apoptosis of the RA FLS cells [117]. Since the RA FLSs have aggressive behavior, like cancer cells, and many investigations have shown benefits from HDAC inhibitors in cancer therapy, these kinds of treatment could be an option in the case of RA. Trichostatin A is one of the important HDAC inhibitors that has been widely used to inhibit the aggressiveness of the RA FLSs [118]. Studies have illustrated that treatment of the RA FLSs with trichostatin A could decrease invasiveness [113] and inflammatory responses [119] as well as increase apoptosis [113, 120–122]. The low proliferation of the FLSs could be mediated by inhibition of cell-cycle progression, and the high apoptosis could be applied through high expression of pro-apoptotic genes [123]. In vitro studies have shown that combination therapy of SAHA and MS-275, histone deacetylase inhibitors, could prevent the proliferation of the FLSs [124]. Furthermore, HDACs have lots of non-histone targets such as *P53*, a critical gene in regulating cell proliferation and apoptosis, which is downregulated through HDACs [125]. Investigations on animal models of RA have shown that general HDAC inhibitors could decrease disease severity [124, 126, 127]. Furthermore, they have documented that HDACs play a critical role in RA pathogenesis by modifying the FLSs.

### MicroRNA abnormalities

In respect to microRNAs in the RA FLSs, a recent study has shown that there is a different signature of microRNA expression in the RA FLSs compared to the OA FLSs. Of these, the expression of miR-10a in the RA synovial tissue and the FLSs was significantly lower than the OA FLSs. Pro-inflammatory cytokines such as IL-1b and TNF result in decreased expression of miR-10a through the nuclear factor (NF)-κB pathway. This microRNA targets various genes in the TNF/IL1 signaling pathway include BTRC (beta-transducin repeat containing E3 ubiquitin-protein ligase), nuclear receptor subfamily 2 group C member 2 (NR2C2), and interleukin-1 receptor-associated kinase 4 (IRAK4) [128]. Upregulation of miR-10a decreases the expression of C-C motif chemokine ligand 2 (CCL2), matrix metallopeptidase (MMP)-1, MMP-13, IL-6, and IL-8 and reduces migration, proliferation, and invasion of the RA FLSs [129]. Another microRNA with a reduced expression profile is miR-27a, which its expression is highly downregulated in FLSs, synovial tissue, and serum of RA patients compared to healthy controls. This study further illustrated that mimic of miR-27a could markedly inhibit invasion and migration of the RA FLSs through downregulating of MMP-2/9/13 and invasion-related proteins such as RhoA, Rac1, and Cdc42. Furthermore, miR-27a reduce NF-kB p65 and TLR4 protein levels. Another molecule that is regulated by miR-27a is follistatin-like protein 1 (FSTL1), a pro-inflammatory mediator with high expression pattern in RA serum and synovium, especially in patients with positive anti-citrullinated protein antibodies (ACPA) [130]. Furthermore, upregulation of miR-27a could increase invasion and migration of the RA FLSs through downregulating the FSTL1, which consequently led to inhibiting of the NF-*κ*B signaling pathway in the RA FLS [131].

A study has shown that the miR-30a expression is reduced in RA patients, compared with healthy controls. Due to miR-30a interactions with Beclin-1, autophagy marker, it was linked to autophagy [132]. Since the miR-34a has been suggested as a regulatory factor for apoptosis, therefore it is linked to the RA FLSs lifespan. Evaluation on miR-34a expression and apoptosis by Niederer et al. has illustrated that the miR-34a targets X-linked inhibitor of apoptosis protein (XIAP). They have documented that there is a negative correlation between high expression of XIAP and low expression of miR-34a in RA FLSs and eventually low expression of miR-34a could lead to apoptosis resistance [133]. In the case of apoptosis, downregulation of miR-15a in the RA FLSs has been reported. Since miR-15a directly targets B cell lymphoma 2 (Bcl-2), which is associated with cell survival, its regulatory role in suppressing Bcl-2 ultimately leads to increased FLS apoptosis in the collagen-induced arthritis (CIA) mice model [134]. Gao et al. have documented that targeting phosphoinositide-3-kinase regulatory subunit 2 (PIK3R2) by miR-126 may modulate apoptosis of the RA FLSs through interfering with the PI3k/AKT signaling pathway [135].

It has been documented that the cyclin-dependent kinase 2 (CDK2) expression could be regulated by the miR-124a, which is associated with MCP-1 secretion and synoviocyte proliferation [132]. Later studies have evaluated the methylation profile of the promoter region of miR-124a in the OA and RA synoviocyte tissue and illustrated that the promoter region in the RA patients was significantly hypermethylated, compared with the OA patients. Hypermethylation of miR-124a might be correlated with its low expression [136], which is correlated to a high proliferation of synoviocytes [137]. Besides, miR-146a can act as a key epigenetic regulator of inflammation in RA, and in regulating the metabolic activity of synovial fibroblasts and their bone destruction [138]. Downregulation of miR-155 in the RA FLSs leads to proliferation and high production of MMP-3. Therefore, overexpression of miR-155 results in decreased MMP-3 production, proliferation and invasive behavior; so, miR-155 could be a therapeutic target [136]. Nonetheless, miR-155 has various functions and implicated in many immune regulatory mechanisms such as; T cell-dependent inflammatory responses, T cell-dependent antibody responses, Treg suppressive functions, B cell development, and activation of tissue macrophages; therefore, it is hard to propose it as a therapeutic marker. One of the validated targets for miR-155 is inositol polyphosphate-5-phosphatase D (INPP5D), which has an inhibitory effect on the proliferation of lymphoid and myeloid cells; therefore, the deregulation of miR-155 might lead to fibroblast proliferation and resident myeloid cell infiltration [139]. The analysis showed that the miR-221 silencing could decrease the invasion and migration of the RA FLSs [140].

High expression of miR-663 increases various key disease targets during the RA development and finally leads to the high proliferation of the RA FLSs [141]. Investigations have illustrated that the miR-21 silencing in the RA FLSs is associated with low expression of NF-κB and also the low proliferation rate of the RA FLSs. On the other hand, high expression of miR-21 was linked with the high level of NF-κB and the elevated level of proliferation in the RA FLSs. Further investigations have illustrated that miR-21 through facilitating NF-κB translocation and affecting the NF-κB pathway could increase the proliferation of the RA FLSs [142].

### LncRNA abnormalities

Long non-coding RNAs (lncRNAs) are RNA molecules containing further than 200 nucleotides in length [143]. lncRNAs play a role in the regulating of genes transcription in several ways, such as RNA sponging, alternative splicing, and epigenetic mechanisms [144]. Aberrant expression of lncRNAs has been reported in FLSs from RA patients and that of healthy individuals that were also involved in clinicopathological manifestations of RA patients [128]. Nonetheless, little has been revealed about the involvement of lncRNAs in the cancerous behaviors of RA FLSs. Ye et al. have reported that the silencing of lncRNA ZFAS1 (which is upregulated in RA FLSs) leads to the reduced migration and invasion in RA FLSs. They have reported that lncRNA ZFAS1 is involved in the migration and invasion of RA FLSs through regulating of miR-27a transcription [145]. Another lncRNA, metastasis-associated lung adenocarcinoma transcript 1 (MALAT1), has been reported to play a role in the apoptosis of RA FLSs via repressing the PI3K/AKT signaling [146]. Hence, lncRNAs are involved in the modulation of genes with the implication in the cancerous behaviors of RA FLSs. Altogether, this is a nascent field of research that needs to be enrichened with comprehensive data to let us come up with the understanding of the decisive implication of lncRNAs in the cancerous behaviors of RA FLSs, for designing future diagnostic tools as well as therapeutic approaches.

## Concluding remarks

Interactions between the structural components of the synovial environment, such as synoviocytes with different cells of immune system, such as T lymphocytes, may play a pivotal role in the ethiopathogenesis of RA disease. Meanwhile, in recent years, FLSs have been introduced as the chief cells involved in the initial onset, the development and spread of synovitis, and, ultimately, the destruction of the adjacent joint and bones. Active and transformed RA FLS at the synovial site, with high proliferation, resistance to apoptosis, invasion, metastatic behavior, and induction of angiogenesis, play a cancerous role in RA pathogenesis. The phenotype and cancer-like behavior of FLSs in RA remind the behavior of CAFs in various neoplasms. This study reviewed the different variations of FLSs that led to their cancer-like behavior (in the metabolism, genetics, and epigenetics areas).

Today, the fundamental role of FLSs in the development of synovial hyperplasia, chronic inflammation, invasion, and articular cartilage destruction has been identified. The metabolic, genetic, and epigenetic alterations involved in the transformation of these cells are also somewhat determined. To better understand the behavior of RA FLS in the inflammatory milieu, further studies are required, focusing on similarities to cells that form the cancerous microenvironment, in particular, CAFs. It is hoped to take a fundamental step toward achieving a targeted and effective therapeutic strategy, with a clear understanding of the behavior of RA FLS and its main contributing factors.

## Data Availability

Not applicable.

## Abbreviations

ACPA: Anti-citrullinated protein antibodies
AMPK: AMP-activated protein kinase
Bcl-2: B cell lymphoma 2
BMR: Basal metabolic rate
BRAF: B-Raf proto-oncogene, serine/threonine kinase
BrPa: Bromopyruvate
BTRC: Beta-transducin repeat containing E3 ubiquitin-protein ligase
CAFs: Cancer-associated fibroblasts
CCL2: Chemokine ligand 2
CDK2: Cyclin-dependent kinase 2
CK: Choline kinase
CVD: Cardiovascular diseases
DAF: Decay-accelerating factor
DML: Differentially methylated loci
Dyrk1A: Dual-specificity tyrosine-regulated kinase 1A
EAMs: Extra-articular manifestations
FGF: Fibroblast growth factor
FLS: Fibroblast-like synoviocyte
FSTL1: Follistatin-like protein 1
G6PI: Glucose 6-phosphate isomerase
GLUT1: Glucose transporter 1
HDACs: Histone deacetylase
HK2: Hexokinase 2
IHC: Immunohistochemistry
IL: Interleukin
INPP5D: Inositol polyphosphate-5-phosphatase D
IRAK4: Interleukin-1 receptor-associated kinase 4
JAK: Janus kinase
LBH: Limb bud and heart development
LINE1: Long interspersed element 1
lncRNAs: Long non-coding RNAs
MALAT1: Metastasis-associated lung adenocarcinoma transcript 1
MAPK: Mitogen-activated protein kinase
MLS: Macrophage-like synoviocytes
MMP: Matrix metalloproteinases
MMP: Matrix metallopeptidase
mTORC1: Mammalian target of rapamycin complex 1
NF: Nuclear factor
NR2C2: Nuclear receptor subfamily 2 group C member 2
OA: Osteoarthritis
PB: Peripheral blood
PCNA: Proliferating cell nuclear antigen
PDGF: Platelet-derived growth factor
PDGF: Platelet-derived growth factor
PI3K: Phosphatidylinositol-3-kinase
PIK3R2: Phosphoinositide-3-kinase regulatory subunit 2
PTPN11: Tyrosine-Protein phosphatase non-receptor type 11
RA: Rheumatoid arthritis
SAPK: Stress-activated protein kinase
SCID: Severe combined immunodeficiency
SF: Synovial fibroblasts
Spry2: Sprouty RTK signaling antagonist 2
TCA: Tricarboxylic acid
TGF-β: Transforming growth factor-beta
TNF-α: Tumor necrosis factor-α
UDPGDH: UDP-glucose 6-dehydrogenase
VCAM-1: Vascular cell adhesion molecule-1
VDAC: Voltage-dependent anion channel
VEGF: Vascular endothelial growth factor
XIAP: X-linked inhibitor of apoptosis protein
